# Abstract of Stenographer’s Report, U. S. V. M. A.

**Published:** 1896-03

**Authors:** 


### SOCIETY PROCEEDINGS.

### ABSTRACT OF STENOGRAPHER'S REPORT, U. S. V. M. A.

(Continued from page 70.)

THE second day's session convened with a much larger attendance than the day previous. The name of Veterinarian Desmond, of Warnambool, Victoria, was recommended for honorary membership by Drs. Harrison and Stewart and referred to the *comitia minora.*

The Secretary was requested to read a letter from Dr. L. McLean relative to the Prize Committee, and asking that he be relieved from any further duty in connection therewith. Dr. Lyman, a former Chairman of the Prize Committee, referred to the fact of no papers being submitted during his term of appointment.

The report of the *comitia minora* was read and received, and their recommendations as to membership were approved.

The charges of violation of code of ethics against Dr. Geo. H. Burns were sustained, and it was agreed that he be given an opportunity to remove said violation.

The communication from the Acting Secretary of the Association of Faculties relative to the open letter of Prof. Liautard was received, and their recommendation, that a joint committee of the two Associations draw up a suitable reply, was concurred in. After which a motion that said committee consist of Drs. Hoskins, J. C. Meyer, Jr., and W. L. Williams was adopted.

Section E, of the report of Committee on Diseases, was called for, and Dr. S. J. J. Harger responded, dwelling upon glanders and the best methods of extermination, the use of mallein, etc. Incidentally he referred to the various infectious and contagious diseases prevalent in Pennsylvania.

Section C, which covered Southern cattle fever, had been assigned to Dr. S. S. Dinwiddie, in whose absence the report was read by Chairman Trumbauer.

The President declared the report open for discussion. Dr. Williams responding, referred to the criticism of his statements as to the existence of glanders in the Rocky Mountains. Alluding to the mild form which often prevails, he also outlined three very acute cases, with great structural changes, which had come under his observation, and most emphatically contradicted the inference made in the report that these were chronic cases of malignant catarrh. He recorded satisfactory results with mallein as a diagnostic agent. He also referred to spasm of the glottis, which frequently occurred in epizoötic form. He criticised the great length of the report of the Committee on Diseases, particularly as a similar length of time could not be accorded for its discussion.

The five-minute-rule for discussion was, on motion, adopted. A motion first prevailing that the discussion of that part of the report on tuberculosis be postponed until the other papers on that subject be read; carried.

Dr. Mayo criticised the lax manner that prevailed for the suppression of glanders and how it was allowed to spread through the concession to ignorant pretenders who claim to cure these cases.

Dr. Salmon thought that the claim that the true germ of contagious pleuro-pneumonia had been discovered was not to be accepted too hastily, as others who had most thoroughly investigated this subject had failed. As to Texas fever, he believed it was spread by other channels besides the ticks. He believed that Southern cattle could hardly at any time be considered immune, as one at Washington, after six years' isolation from its native heath, still retained in its blood propagating powers by which the disease was reproduced experimentally.

Dr. T. J. Turner, in speaking of his con version to acceptance of the tick-theory, said that the Board of Agriculture, of Missouri, instruct their sanitary officers to quarantine where the ticks are found.

Dr. Cary asked the question if it would not be practicable to allow these Southern cattle to be removed into Northern districts that the ticks might be removed.

Dr. Salmon, in reply, admitted the possibilities of this plan, but referred to the great difficulty of removing all the ticks, and that dips now recommended had in some instances killed the cows as well as the ticks. Further experiment in this direction was under investigation.

The chemical nature of the dips being asked for, it was stated that usually crude carbolic acid was the basis, though almost everything had been tried, experimentally, in the laboratory.

Dr. Peters referred to some dogs reported to have been affected with an anthracoid form of disease, but which laboratory research failed to find the bacillus anthracis, but in all of the cases a belted germ was observed.

Dr. A. H. Baker, who had reported these cases, first stating that it was not confined to any one breed, was not infectious, the animals were not disposed to bite one another, no inclination to gnaw, that the jaw drops, tongue protrudes, is of a dark color, the disease terminating fatally in from four to twenty-five days. He did not consider it rabies. The head of the Pasteur Institute at Chicago considers it a form of rabies.

Dr. Peters said in Nebraska the disease was confined to bird dogs, and he believed it infectious.

Dr. Faust believed it to be the dumb or mute form of rabies.

Dr. A. H. Baker believed there was no rabies without delirium and a disposition to gnaw and bite.

Dr. Lyman reported an outbreak in Boston of a similar type, and, after laboratory investigation by Dr. Ernst, the conclusion reached was that it was dumb rabies.

Dr. Salmon said the diagnostic point of rabies is the possibility of transmitting the disease by reason of contact, after the mode of Pasteur. If you have a disease you can transmit it to a rabbit or other small animal, and it runs through the typical course of rabies in those animals; so far as we know from the present condition of that disease, it must be rabies.

Dr. Lyford, after a number of experiences, in many of which he endeavored to assign causes for the symptoms exhibited, and on which many autopsies were held with varying unsatisfactory lesions, concluded that these cases were a form of rabies.

Dr. Pearson referred to many cases seen in Philadelphia, and of similar symptoms, such as foreign bodies in the stomach of those destroyed in the furious form and those succumbing to the mute form. Inoculations into the brain of rabbits brought practically the same symptoms as those diagnosed as dumb rabies.

Dr. Schwarzkopf wished to take exception to the disposition of some to consider this disease allied to diphtheria, as the latter seemed to be only an affliction of the human race.

Dr. Walrod reported a case of rabies in a cow, supposed to have originated from a bite from a dog, the latter having been bitten in a fight while away from home by a strange dog, the dog having died suddenly with obscure symptoms.

Drs. Brenton and Meyer also reported similar experiences of cases they termed dumb rabies.

Dr. T. J. Turner, speaking of the subject of anthrax, reported the outbreak in Missouri, where six cows, three mules, twenty-two hogs, and one sheep succumbed to the outbreak. The first cow, dying suddenly, was hauled out by the owner and skinned and fed to the hogs. The farmer became inoculated in handling the carcass. The disease had been transmitted to this farm through a city sewer which passes through the premises. Vaccination with anthrax lymph was under adoption, with favorable results so far.

After a brief reference to actinomycosis and usages in condemnation at the Union Stock Yards at Chicago, on motion the discussion closed.

The report of the Prize Committee being called for, Chairman Dr. Tait Butler responded with an individual report, as no papers had been offered, and, pending the filing of such, he had delayed conferring with his colleagues, and felt that this explanation was due the convention and in justice to Dr. L. McLean, one of the members of the committee.

The report, on motion, was accepted, and the President asked what action would be the pleasure of the convention as to the recommendations made.

On motion, the convention concurred in the recommendations of the Chairman.

The Secretary, at the request of the Chair, read, in the absence of Chairman Wm. Dougherty, the report of the Committee on Act of Incorporation.

The Committee had been unable to secure favorable action on the proposed measure, and, on motion, the report was received and the Committee continued, as it was special in character.

The Secretary, at the request of the President, read the report of Chairman J. P. Turner, of the Army Legislative Committee. The report bore a very sanguine and hopeful aspect for successful termination of this long and trying struggle for recognition at the hands of the incoming Congress. On motion, the report was accepted. The question of furnishing the Committee with funds in the face of an empty treasury was referred to the *comitia minora*.

The Publication Committee, owing to the low condition of the funds of the Association, reported the inability of the Committee to publish the proceedings of the meeting for 1894. Dr. Osgood, referring to the deficit, thought it would be well for the Association to be better informed as to the cause of this deficit. The President responded by saying that the Association had about four hundred members, that the annual dues were $2.00, that the publication of the proceedings for '92 and '93 had cost each year, when delivered, over $800.00, thus the Association was handing back to its members more than received from them, besides leaving no money for committees, officers' expenses, etc. Hence, the resolution adopted at Philadelphia to make the annual dues, for and after 1895, five dollars per year.

Dr. Williams in a brief manner referred to the importance of the work of the Finance Committee, and also the Publication Committee, and said greater care should be exercised in their selection.

The Secretary's report followed, which referred to the arduous duties of this post and the difficulties labored under during the year just closed.

With the exception of the State Secretary's report for Massachusetts, the other State reports were read by title and referred to the Publication Committee. By special request that of Massachusetts was read. As the report dealt specially with tuberculosis, its discussion was laid over for consideration in conjunction with the latter subject.

Resolutions on the deaths of members Bryden, Leis, Paaren, and Middleton were read and adopted.

The afternoon session convened at 3 o'clock, and, after some preliminary work, the election of officers was reached, resulting in the following choice for the ensuing year: President, W. Horace Hoskins, Philadelphia; Eastern Vice-President, F. H. Osgood, Boston; Central Vice-President, C. C. Lyford, Minneapolis; Western Vice-President, R. H. Harrison, Atchison; Secretary, S. Stewart, Kansas City, Kansas; Treasurer, James L. Robertson, New York City.

The President then called for new business and referred to the criticism made of the length of time allowed committee reports, and asked for an expression of opinion of the convention on the subject.

Dr. Harger, referring to the necessity of a limit of time to committee reports, suggested a committee to review these reports, to abstract them, bringing out the leading features of each report; another committee to take in charge the papers; also, that members should be designated to discuss salient features of reports and papers.

Dr. Osgood thought that most chairmen and those preparing papers would object to having the same altered by a committee. He thought the best plan would be a division of time and the allotting of specified periods of time for each report, paper, etc.

Dr. Mayo, in agreeing with the views of the preceding speaker, moved that the reports of the various committees be limited to thirty minutes.

Dr. Harrison suggested the referring of reports of committees, Corresponding and State Secretaries' reports, without reading, to the Publication Committee, and they to select any salient features desirable to be brought before the Association, and thus grant more time for the consideration of papers.

Dr. Faust favored the time-limit suggested by Dr. Osgood.

Dr. Salmon, in complimenting the excellent work done on the committee reports presented, regretted the lack of time to take up and properly discuss the many subjects elaborated upon. He thought a half or three-quarters of an hour should be allowed for these reports, and that the several subjects should be read and discussed singly.

Dr. Williams thought it was essential that members should know in advance what limit of time was to be allowed the different subjects on the programme; that each year the importance of committees would differ and the personnel would also be a factor to be considered. He thought this matter would be safer in the hands of the *comitia minora*, where reasons or appeals for greater time could be filed as occasion or circumstances demanded. He thought a maximum limit of time might give license to a minor committee, and closed by making a motion to delegate this power to the *comitia minora*.

Dr. Cary asked whether it would not be feasible in limiting time to important committees when important matter was left out or hurriedly reviewed to have the same referred to the Publication Committee.

The motion to place this matter in the hands of the *comitia minora* was seconded and carried.

Dr. Williams then referred to the necessity of granting the Publication Committee power to abstract reports, etc., so as to restrict the great cost of publishing our transactions.

Further discussion of the subject was closed by the Chair and the subject of tuberculosis announced.

The first paper offered on the subject was by Dr. W. B. Niles, of Ames, Iowa. After its reading the President, on behalf of the Iowa veterinarians, extended a cordial invitation to all in attendance to join in accepting the pleasure of a theatre party in the evening.

The paper was then opened for discussion, together with the report of the State Secretary of Massachusetts.

Dr. Mayo reported a case where marked reaction occurred at two periods five and a half months apart, and autopsy failed to reveal tubercular lesions.

Dr. Niles desired to know if injection of tuberculin in mild cases had any tendency to make the disease more acute.

Dr. Reynolds reported results in two herds where injections were kept up for a number of weeks after reaction had led to condemnation, and from the slight lesions revealed on autopsies he was fully convinced that tuberculin in the bovine species did not increase the disease. In one case where extensive lesions were found the animal had maintained an extremely healthy and vigorous appearance up to the hour of her destruction.

Dr. Niles reported the experience of a physician in Iowa in using tuberculin as a curative agent in five cases; in two cases result reported as very satisfactory, one barely satisfactory, and the other two doing fairly.

